# A novel mutation causing nephronophthisis in the Lewis polycystic kidney rat localises to a conserved RCC1 domain in Nek8

**DOI:** 10.1186/1471-2164-13-393

**Published:** 2012-08-16

**Authors:** John K McCooke, Rudi Appels, Roberto A Barrero, Alice Ding, Justyna E Ozimek-Kulik, Mathew I Bellgard, Grant Morahan, Jacqueline K Phillips

**Affiliations:** 1 Centre for Comparative Genomics, Murdoch University, Perth, WA, 6150, Australia; 2 Australian School of Advanced Medicine, Macquarie University, Sydney, NSW, 2109, Australia; 3 The Western Australian Institute for Medical Research, University of Western Australia, Perth, WA, 6000, Australia

**Keywords:** Cilia, Directed next generation sequencing, Electron microscopy, Genome capture, Immunohistochemistry, Nek8, NPHP, Polycystic kidney disease

## Abstract

**Background:**

Nephronophthisis (NPHP) as a cause of cystic kidney disease is the most common genetic cause of progressive renal failure in children and young adults. NPHP is characterized by abnormal and/or loss of function of proteins associated with primary cilia. Previously, we characterized an autosomal recessive phenotype of cystic kidney disease in the Lewis Polycystic Kidney (LPK) rat.

**Results:**

In this study, quantitative trait locus analysis was used to define a ~1.6Mbp region on rat chromosome 10q25 harbouring the *lpk* mutation. Targeted genome capture and next-generation sequencing of this region identified a non-synonymous mutation R650C in the NIMA (never in mitosis gene a)- related kinase 8 ( *Nek8*) gene. This is a novel Nek8 mutation that occurs within the regulator of chromosome condensation 1 (RCC1)-like region of the protein. Specifically, the R650C substitution is located within a G[QRC]LG repeat motif of the predicted seven bladed beta-propeller structure of the RCC1 domain. The rat *Nek8* gene is located in a region syntenic to portions of human chromosome 17 and mouse 11. Scanning electron microscopy confirmed abnormally long cilia on LPK kidney epithelial cells, and fluorescence immunohistochemistry for Nek8 protein revealed altered cilia localisation.

**Conclusions:**

When assessed relative to other *Nek8* NPHP mutations, our results indicate the whole propeller structure of the RCC1 domain is important, as the different mutations cause comparable phenotypes. This study establishes the LPK rat as a novel model system for NPHP and further consolidates the link between cystic kidney disease and cilia proteins.

## Background

Cystic kidney diseases are amongst the most common monogenetic disorders in the human population, affecting up to 1 in 400 individuals [1,2]. Numerous studies have been carried out in humans, zebrafish and rodents [3,4] indicating the important function that cilia and associated proteins play in the pathogenesis of renal cystic diseases [5], including polycystic kidney disease (PKD) [6,7] and nephronophthisis (NPHP) [8] incorporating Merkel Gruber and Joubert Syndromes [9].

In addition to progressing our understanding of cystic pathophysiology, rodent models of cystic kidney disease have played a major role in facilitating the molecular characterization of novel genes required for normal renal epithelial cell function [10,11] and establishing the importance of the ciliary-centrosome interactions in coordinating many aspects of cell division [12]. The available evidence suggests that defects in cilia or centrosomes can have profound effects on the control of cell division. For example, mutation of the *Pkhd1* gene encoding for fibrocystin/polyductin results in autosomal recessive (AR) PKD. This gene was first identified in the PCK rat [13] and encodes an integral structural component of both the cilia and basal body associated with the centrosome [14,15]. Another example relates to polycystin-1 and -2 expression, both of which have been shown to regulate the JAK-Stat pathway, which in turn controls cell cycle arrest in G0 and G1 [16]. When either protein is defective, as occurs in human autosomal dominant (AD) PKD, unregulated cell growth can occur [15,17]. Other key pathways engaged in cellular processes, such as cell cycle regulation, growth and apoptosis, that are regulated by polycystin-1 include those involving Wnt signaling [15]. Wnt signaling has been shown to have oncogenic activity, becoming deregulated in certain types of cancers [18,19] and polycystin-1 regulates genes responsive to Wnt signaling [20], inhibiting proliferation and maintaining normal microtubular structures.

The *NPHP* gene family ( *NPHP1* through to *NPHP12*) encodes a group of proteins that are expressed in the primary cilium, basal bodies/centrosomes or mitotic spindles, and are critical to cell division and cilia function [8,21]. For example Inversin (NPHP2) is thought to act as a molecular switch between the different Wnt signaling pathways and is associated with the accumulation of proteins within the cilia [22,23]. *NPHP1* encodes for nephrocystin-1, and has been localised to centrosomes in interphase and the mitotic spindle pole during mitosis [24], while *NPHP9*, encoding for NIMA (never in mitosis gene a)- related kinase 8 ( *Nek8*), localises to the proximal region of the primary cilium and is proposed to modulate ciliary targeting of polycystin-1 and polycystin-2 [25,26].

The Lewis Polycystic Kidney (LPK) rat is a spontaneous rat model of ARPKD that has been phenotypically characterized [27-29]. The phenotype is localized to the kidney, which presents with large renal cysts. The LPK rat contrasts with the PKD/Mhm cystic rat model, which has a mutation in the *Pkdr1* gene (encoding the protein SamCystin), and which presents additionally with liver cysts [30]. Renal cyst formation in the early stages of disease in the LPK model is confined to the collecting ducts, comparable to human ARPKD, and the animals present with a concurrent and marked hypertensive phenotype [28]. To fully appreciate the pathological basis of progression of the polycystic kidney phenotype in this model, we sought to identify the gene mutated in the LPK rat and its predicted impact on protein function using a positional cloning approach. Further analysis focused on conserved elements within the mutated protein to predict structure and function.

Our results determined that the mutation responsible for the rat LPK model is a non-synonymous mutation R650C in the *Nek8* gene, in a region syntenic to portions of human chromosome (chr) 17 and mouse chr 11. This is a novel Nek8 mutation that occurs within the regulator of chromosome condensation 1 (RCC1)-like region of the protein and is located within a G[QRC]LG) motif that we predict is important for the structural organisation of seven bladed beta-propeller RCC1 like proteins. Scanning electron microscopy confirmed abnormal renal cilia in the LPK and fluorescence immunohistochemistry revealed altered cilia localisation of the Nek8 protein.

## Results

### Genetic analysis locates the *lpk* QTL on rat chromosome 10q25

**Figure 1:**
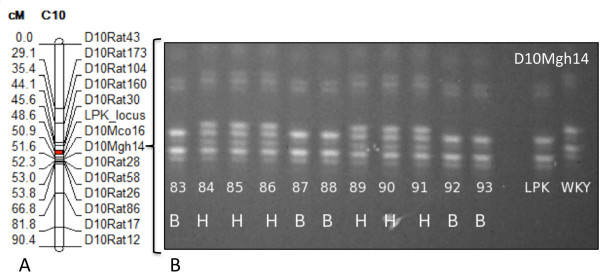
**Localization of the*lpk*locus within chromosome 10.A**. Position of the *lpk* locus (red) relative to polymorphic markers on chromosome 10 (shown in centiMorgan, cM) locations as analysed on the backcross 1 (BC1) generation, scoring the presence or absence of renal cystic phenotype. **B**. Sample gel for marker *D10Mgh14* showing samples scored as homozygote LPK (B), homozygote Wistar Kyoto (A) or heterozygote (H).

**Figure 2:**
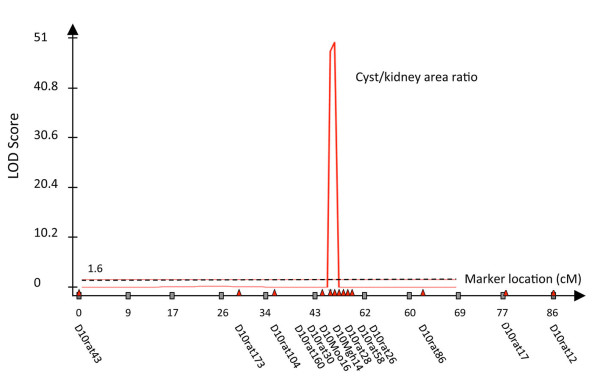
**Locating the*lpk*QTL.** Quantitative trait loci for PKD phenotypic markers were determined using WinCart QTL [67]. The markers used for analysis are shown along the x-axis (red triangles) and span 86cM from D10Rat43 to D10Rat12. On the y axis, the evidence for linkage is recorded as a logarithm of odds (LOD) score. The QTL illustrated is the cyst to kidney area ratio. The QTL was determined using Composite interval mapping with model 6 (standard model) with a window size of 0.5 cM and walk speed of 1cM a forward and backward regression. A threshold of 7.5 (LR) and 1.6 (LOD – black hashed line) was determined by permutations.

Backcross (BC1) progeny were produced and characterized. The genetic map obtained from MapManager [31] analysis of 139 BC1 progeny is shown in Figure 1A. The characteristic LPK phenotype, namely cyst to kidney ratio, was located within the map using WinCart Qtl. The output from WinCart Qtl for marker-trait linkage is shown in Figure 2. The cyst/kidney area ratio quantitative trait locus (QTL) (LPK phenotype) mapped the *lpk* locus with a logarithm of odds (LOD) score of 50 to the region defined by the markers *D10Rat30* (at 63.75Mbp in the rat genome) and *D10Mgh14* (at 65.38Mbp). Positions were taken from the RGSC genome ver3.4 [32]. Characterization of an independent mapping population, a second filial (F2) generation of 152 progeny, confirmed the location of the *lpk* locus but was unable to provide any greater resolution with the peak region of linkage located between *D10Rat30* and *D10Rat161* (at 67.09Mbp).

### Comparative genomics identifies candidate genes

**Figure 3:**
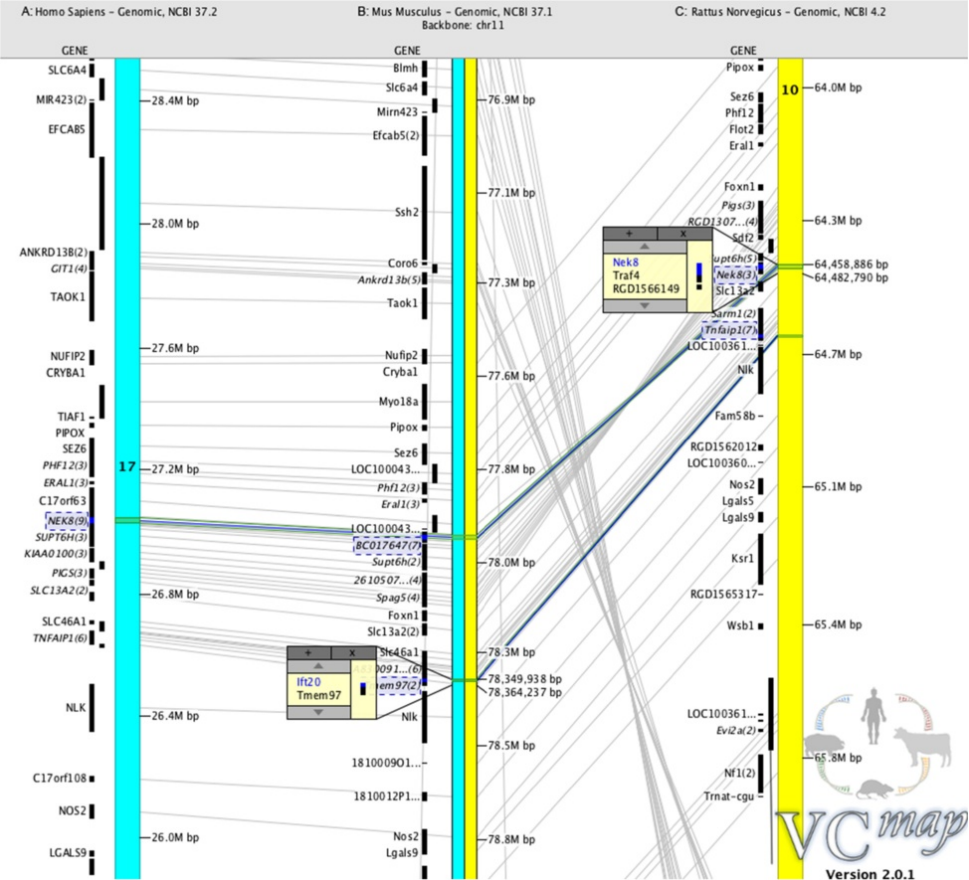
**Human, mouse and rat synteny.** Map of the syntenic relationship between human ( **A**), mouse ( **B**) and rat ( **C**) over the region defined in this study, as generated by VCMap [32] illustrating the annotated genes. A: Homo sapiens: NCBI 37.2, B: Mus muscuus: NCBI 37.1, C: Rattus norvegicus: NCBI 4.2. Genes of particular interest to this study are highlighted.

Localization of the *lpk* mutation on rat chromosome 10 (RRA10) allowed comparison with syntenic regions of mouse and human chromosomes, MMU11 and HSA 17 respectively (Figure 3). The region of interest as defined by the QTL analysis (63.6 to 65.2 on RRA10; Figure 3C), projected onto HSA17 27.6Mbp to 25.8Mbp (Figure 3A) and MMU11 77.5Mbp to 77.8Mbp (Figure 3B), based on shared molecular markers. Within this region we identified 46 syntenic genes. Two of these genes have previously shown to be associated with cystic kidney disease in mammals, namely, the genes coding for the Nek8 protein and for the intraflagellar transport protein 20 (IFT20).

**Figure 4:**
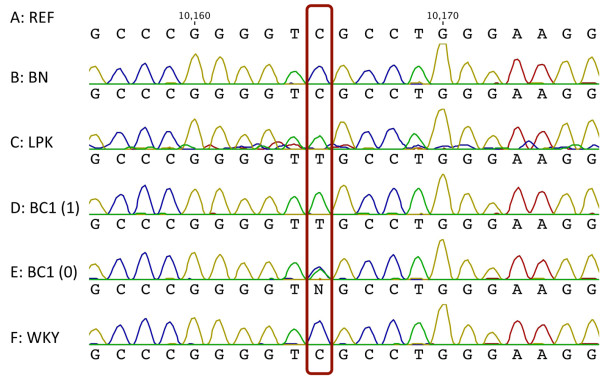
**Sequencing of LPK*Nek8*mutation.** Chromographs show the *lpk* mutation as a SNP within the *Nek8* gene. Lines A and B are the Brown Norway (BN) reference sequence (REF) (NC_005109.2, gene ID: 287473) and the BN parent sequence data; both showed a homozygous Cytosine (C) at the position of the SNP (red box). Lines C and D are the LPK parent and BC1 (1: cystic positive) progeny and contained the homozygous Thymidine (T). Panel E is the BC1 (0: cystic negative) progeny and is heterozygous (C/T). Panel F is the other parental rat strain Wistar Kyoto (WKY) which had a homozygous Cytosine (C) at the position of the SNP.

Following this comparative analysis, several approaches were explored to identify additional genes in the QTL defined region that may be causal for the LPK phenotype. An initial subset of genes was determined by review of all genes in the region, captured using Ensembl Biomart [33] based on qualitative assessment criteria linked to known function and network pathways. Analyses indicated 13 genes, in addition to *Nek8* and *Ift20* within this area that could potentially result in cystic kidney disease, namely: *RGD1309077Phf12, Flot2, Spag5, Sdf2, Supt6h, Proca1, Traf4, Sarm1, Nlk* and *Ksr1* (Additional file 1: Table S1). These candidate genes span approximately 478 kbp of genomic sequence so a genome capture array was employed to sequence the entire *lpk* locus (~1.6Mbp) rather than using gene-by-gene sequencing.

### Targeted genome next-generation sequencing of the *lpk* region

Nimblegen arrays were used for exon capture and sequencing of genomic DNA spanning the markers *D10Rat238* and *D10Rat161* on RRA10 (4,406,155 bp). Totals of 44 million reads and 48 million reads were generated for the LPK sample and control Lewis (LEW/SsNArc) DNA, respectively. 76% of the reads mapped to the target region with an average depth coverage of 500. Reads were aligned to the rn4 rat assembly to identify single nucleotide polymorphisms (SNPs) within exons. Only one single homozygous and 3 heterozygous SNPs (Table 1) were found unique to the LPK strain. The homozygous mutation was located at 64,469,050 bp (chr10) within exon 14 of the *Nek8* gene, consisting of a base change of cytosine to thymine. This corresponded to position 2008 within the cDNA and would cause an arginine to cysteine change at position 650 in the protein product. Conservation analysis identified the relevant amino acid (arginine) to be conserved in published *Nek8* mRNA sequences [34]. The non-synonymous heterozygous SNPs identified in the sequence analysis were in two separate genes. The first was a het-2 mutation (654/700 reads) at location 63385139 bp within the gene Fam101b (family with sequence similarity 101) of A/G (P176L) with a coverage of 654 reads. The second gene contained two low frequency het-1 mutations of T/A (57/152 reads) and A/T (60/155 reads) at location 66106990 bp and 66106990 bp within the *krt1-18* (Keratin, type 1 cytoskeletal 18) gene. Conservation analysis of all three heterozygous SNPs (using *Rattus norvegicusMus musculusHomo sapiensCanis lupisGallus gallus* and *Xenopus tropicalis*) indicated that although the nucleotide in which the SNP occurred was conserved across species, the actual codons were not conserved. These SNPs were not considered further.

**Table 1:** Non-synonymous mutations

Position*	**Ref**	**Obs**	**A**	**C**	**G**	**T**	**total**	**used**	**SNP Type**	**Gene ID (ENSRNOG)**	**Name**	**Description**	**cDNA**	**Prot**	**AA change**
63385139	G	AG	339	1	314	0	714	654	het2	00000006674	*Fam101b*	Protein FAM101B	647	176	P- > L
64469050	C	T	0	0	0	392	393	392	hom	00000012866	*Nek8*	serine/threonine-protein kinase Nek8	2008	650	R- > C
66106990	T	TA	25	0	0	32	152	57	het1	00000022699	*LOC303341*	Keratin, type I cytoskeletal 18	464	155	Y- > F
66106991	A	AT	32	0	0	28	155	60	het1	00000022699	*LOC303342*	Keratin, type I cytoskeletal 19	463	155	Y- > N

List of non-synonymous mutations derived from Nimblegen array target sequencing and bounded by the genes *Myo1c* and *Slc6a4* and using the flanking markers *D10Rat161* and *D10Rat238* [62685555-67091740].

*Position location in base pairs (bp) was determined using the Rat Genome Browser Gateway using the Baylor 3.4/ build rn4 (http://genome.ucsc.edu/cgi-bin/hgGateway?org=rat). Ref: nucleotide on the ref sequence. Obs: nucleotide recorded from transcripts. A C G T: number of transcripts recorded with a change to the reference. Total: total number of transcripts recorded with a change to the reference. Used: reads that support the change after consideration of read quality. SNP type: defines type of mutation. DNA: position of the base change within the cDNA. prot: position of the amino acid change within the protein. AA change: resultant change of amino acid in the protein. Hom: homozygous. Het: heterozygous.

### Confirmation of the mutation in the *Nek8* gene

Four BC1 animals – two affected and two unaffected as defined by kidney histology and genotyping - were analysed in more detail. The parental lines of Brown Norway (BN), Wistar Kyoto (WKY) and the LPK mutant were also analyzed. Sequencing of BN and WKY DNA revealed a homozygous nucleotide [C] at location 64,469,050 bp (chr10) (Figure 4). The BC1 animals that were cystic negative were either homozygous for nucleotide [C] or were heterozygous [C/T]. The animals positive for cysts were homozygous for [T], as was the LPK mutant. These results confirmed the data obtained from the targeted genome sequencing.

### Nek8 predicted protein structure

**Figure 5:**
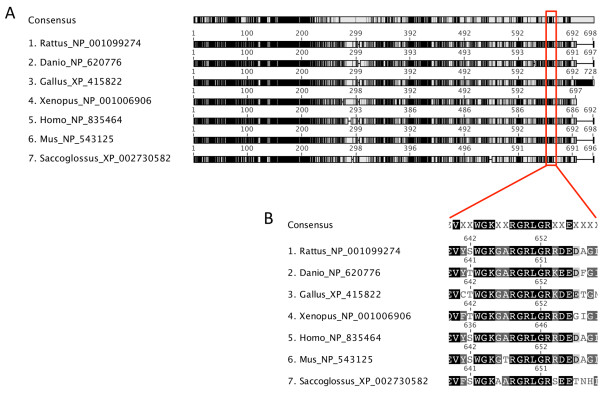
**Location of Nek8 mutation and species sequence alignment.** Sequence alignment of Nek8 protein from 7 representative species, created from known *Nek8* mRNA sequences with the MAFFT algorithm [77]. Panel A illustrates the complete Nek8 alignment. Black indicates 100% conservation scaled to white (0% conservation). The location of the R650C mutation is highlighted within the red box. The serine/threonine kinase region (N terminal) shows high conservation while the alpha tubulin suppressor domain region of the protein (C terminal) shows less conservation and contains the regulator of chromosome condensation-1 (RCC1) domains. Panel B shows the area of the R650C mutation in greater detail to illustrate the conservation of the G[R]LG across the species. The R650C lies within a RCC1 domain. Species represented are the Rattus (rat), Danio (zebra fish), Gallus Gallus (rooster), Xenopus (frog), Homo (human), Mus muscularis (mouse) and Saccoglossus (acorn worm) and their respective accession numbers.

The location of R650C within the Nek8 protein is shown in Figure 5. Alignment of the Nek8 protein sequence across 7 species indicated a high level of conserved residues within the N-terminal serine/threonine kinase region of the protein as compared to the C-terminal RCC1 domain (Figure 5A). The R650C located to a conserved RCC1 like repeat domain containing the repeated motif G[QRC]LG (Figure 5B). The predicted ancestral DNA sequence with evolutionary conserved amino acids in the Nek8 protein is shown in Additional file 2: Figure S1, and suggests that R650C mutation is located within an evolutionary conserved domain.

**Figure 6:**
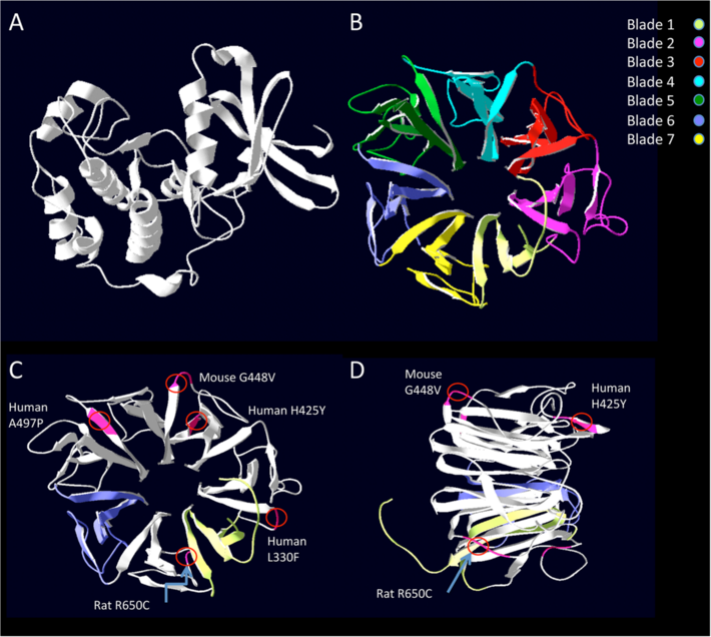
**Phyre2 predicted 3D representation of Nek8 and location of known and R650C mutations.** Panels illustrate the predicted tertiary protein structure for the Nek8 protein, by homologous threading by Phrye2 [37] . Panel A illustrates the Nek8 serine/threonine region and panel B illustrates the RCC1 domains. The colors indicate our annotated blade structures that make up the well-established propeller structure. Panels C and D show the RCC1 domain in two perspectives, illustrating the spatial organisation of known mutations for NPHP9 in human, mouse (*jck*) and R650C (illustrated by the red circles). Numbers indicate the standard designation of mutation within the protein. For example, Human A497P indicates the alanine at position 497 has been mutated to proline. Figures highlight the peripheral location of the mutations, indicating the probable requirement of all blades in protein interactions.

The overall structure of the Nek8 protein was subsequently interpreted using the extensively studied RCC1-like domains in proteins [35,36] as a model. Homology alignment using Phyre2 [37] and ESyPred [38] showed that the serine/threonine kinase at the N terminal of the Nek8 protein had up to 40% homology to over 100 different kinases (data not shown). The RCC1 domain in the C terminal half of the protein aligned with three proteins, PRP20P and RCC1/BLIP and Herc2, which were subsequently used to predict the tertiary structure of the Nek8 protein. The Nek8 RCC1 domain comprised of 6 identifiable RCC1-like repeat domains (Additional file 3: Figure S2). An additional RCC1-like repeat domain was annotated as a non-canonical domain as it did not contain the typical repeat motif. The 3D structure shown in Figure 6 provides a model for the serine/threonine kinase and the RCC1 domains of Nek8.

A manual comparison of the predicted 3D structure of Nek8’s RCC1 domain to the *Mesocricetus auratus* (Golden hamster) [39] confirmed that repeat regions within the RCC1 domain formed the blades of a classical propeller structure [36,40]. The R650C mutation in the LPK Nek8 protein is located in blade 7 (Figure 6C).

### Relative occurrence of G[QRC]LG within proteins

The R650C mutation is within the core G[QRC]LG motif that was also noted within the hamster RCC1 protein. To investigate the occurrence of this motif and possible protein function, the Expasy ScanProsite tool [41] was used to scan for the sequence within UniProtKB/Swiss-Prot databases. The original search using the G[QRC]LG motif within the homo sapiens taxa yielded 966 proteins. The final sequence search was refined to contain the two conserved Glycine residues upstream from the motif of interest. The final search motif was GX(5)GX(4)G[QRC]LG. This search identified 23 proteins: DACT3, FBX24, GLI1, HERC1, HERC2, HERC3, HERC4, HERC5, HERC6, IBTK, MYCB2, Nek8, Nek9, PTF1A, RCBT1, RCBT2, RCC1, RCC2, RCCD1, RPGR, SRGEF, WBS16, WDFY4. Of the 23 identified proteins, five [SGREF, RPGR, RCC2, RCC1, and RCBT2] were characterized as guanine releasing factors, DACT3 and GLI1 are involved in the Hedgehog and Wnt signaling pathways, 8 proteins were associated with the ubiquitin pathway [Herc1 to 6, FBX24, MYCBP2], 5 proteins were linked to cell cycle regulation [Nek8, Nek9, RCBT1, RCC1 and RCC2], while little is known about WBS16, WDFY and RCCD1. The majority of the proteins (18/23) contained multiple copies of the RCC1 repeats and are expected to form toroid 3D structures important for protein-protein interactions.

### Characterization of cilia and Nek8 expression in LPK renal epithelial cells

**Figure 7:**
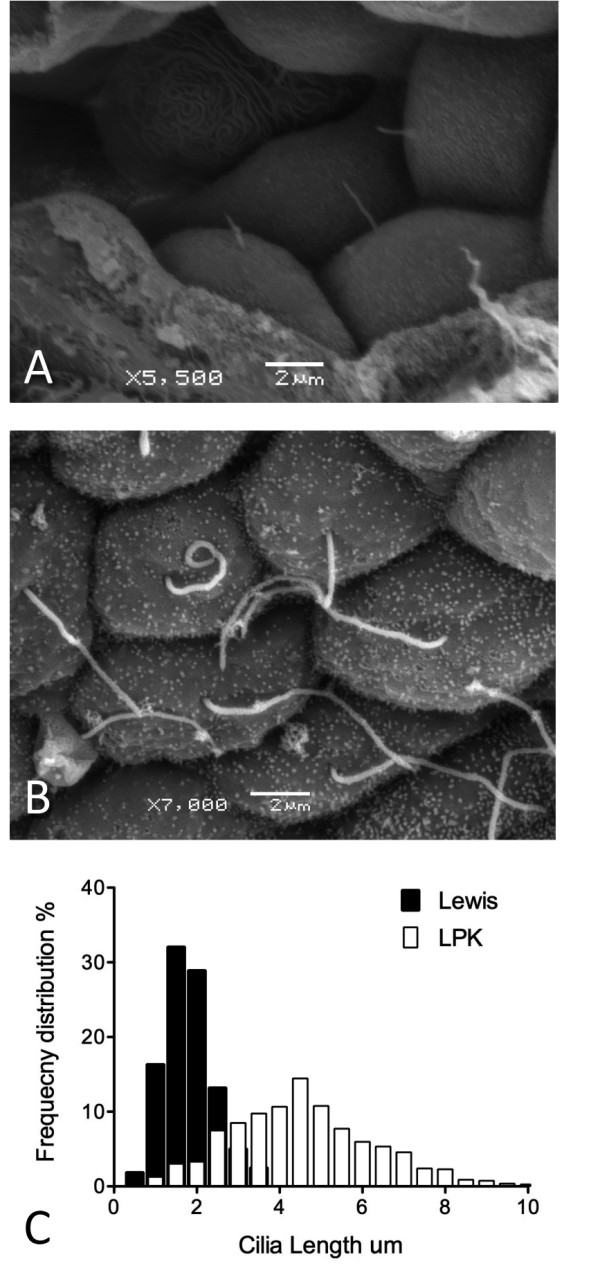
**Determination of cilia structure in the LPK.** Scanning electron micrographs of renal epithelial cells from Lewis control ( **A**) and LPK ( **B**) kidney. The median length of primary cilia is greater in the LPK and they show a greater range of cilia lengths ( **C**). Scale bars in panels A & B are indicative for that panel.

**Figure 8:**
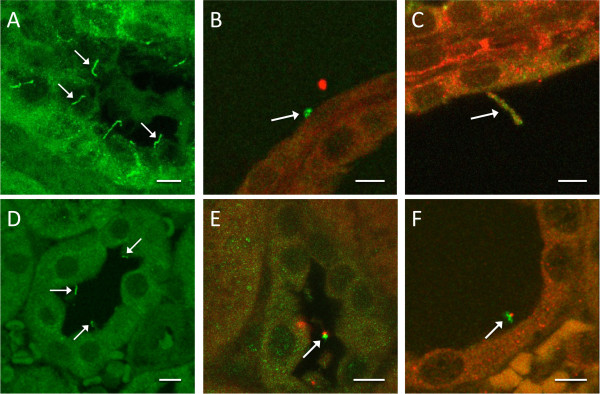
**Localisation of Nek8 in the cilia of Lewis and LPK.** Immunohistochemical labeling for acetylated α-tubulin, γ-tubulin and Nek8 in LPK ( **A**, **B**, **C**) and Lewis ( **D**, **E**, **F**) animals. Single labeling for acetylated α-tubulin (panels A and C) confirmed the abnormal length of cilia in the LPK animals. Double labeling with Nek8 (red) and γ-tubulin (green, as a marker for the basal body) indicated that Nek8 was in close proximity to the base of the cilia in the Lewis ( **E**), but was located at variable positions along the cilia in the LPK ( **B**). Double labeling with Nek8 (red) and acetylated α-tubulin (green, panels C and F) confirmed the altered expression of Nek8 along the length of the cilia in the LPK ( **C**). Arrows highlight examples of cilia identified by either acetylated α-tubulin or γ-tubulin. Scale bars in each panel represent 5μm.

**Figure 9:**
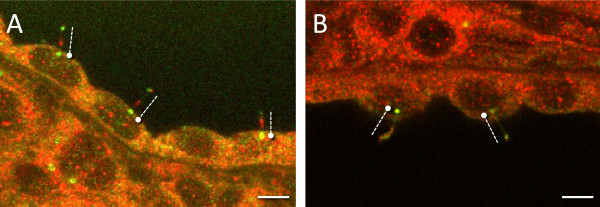
**Abnormal expression of Nek8 along the cilia in the LPK.** Immunohistochemical triple labeling for acetylated α-tubulin, γ-tubulin and Nek8 in LPK animals. The use of both γ-tubulin (green, as a marker for the basal body) and acetylated α-tubulin (green, as a marker for the cilia) confirmed the altered expression of Nek8 (red) along the length of the cilia in the LPK. Two examples illustrating multiple cilia are presented in panels A and B. Round circle indicates γ-tubulin while the dashed line highlights the cilia as identified using acetylated α-tubulin. Scale bars in each panel represent 5μm.

Under scanning electron microscopy, control Lewis rat kidneys had normal morphology, with cilia present on the majority of cells (excluding intercalated cells of collecting tubules) as short projections into the unchanged lumens of the tubules (Figure 7A). Cilia in LPK kidneys were observed as long, often tangled, screwed or with branches or knots (Figure 7B) and there were occasionally multiple cilia per cell. In other cysts, epithelia had no or very short cilia. The median and quartile length of the cilia in the LPK distal tubule and collecting duct was significantly longer than Lewis (LPK: 4.4 μm [3.3/5.6] vs. Lewis: 1.7 μm [1.4/2.2], P < 0.0001), with the range of values notably greater in the LPK kidney (Figure 7C). Immunohistochemical labeling for acetylated α-tubulin confirmed greater ciliary length in the LPK vs. Lewis (Figure 8A and 8D). In the Lewis, double labeling for Nek8 and γ-tubulin, or Nek8 and acetylated α-tubulin, indicated that Nek8 was localised to a discrete region in the proximal portion of the cilia (Figure 8E and F). In the LPK, double labeling with Nek8 and γ-tubulin, or Nek 8 and acetylated α-tubulin showed variable positioning of Nek8 in cilia, being either absent, present at the base or distal region of the cilia, or showing punctate staining along the length of cilia (Figure 8B and C). This was confirmed in additional experiments with triple labeling for Nek8 and both γ-tubulin and acetylated α-tubulin (Figure 9).

## Discussion

This study found a novel SNP mutation (R650C) located within the Nek8 gene on rat Chr 10 is responsible for the autosomal recessive cystic kidney disease phenotype identified in the LPK rat [28]. The LPK rat can now be classified as a rat model of NPHP9 [21], and will be invaluable for delineating the complex disease processes associated with NPHP, including hypertension and cardiac disease [42].

The LPK phenotype is similar to that described for the *jck* mouse Nek8 model [7,43], which also have elongated renal cilia and altered ciliary Nek8 expression [26,43,44]. Longer cilia have also been reported in other animal models of cystic renal diseases. The collecting ducts of the *wpk* rodent model for Meckel-Gruber syndrome [45], and NPHP3 mice [46], all display longer cilia. An increase in length variability has been described previously in the *cpk* mouse [47], a model of ADPKD. Our data supports the hypothesis that ciliary Nek8 plays a key role in normal communication between the cilia and the processes of cellular differentiation and proliferation, such that its reduced expression or inadequate localization results in the formation of cysts [7]. Whether an increase in cilia length is the effect of cystogenesis, or a primary defect in the assembly and maintenance of the cilium as a key pathogenic mechanism remains a controversial issue, as elongation of the cilium is also linked to injury [48-50].

The impact of an arginine (R) to cysteine (C) change can potentially result in disulfide bridge formation, resulting in loss of rigidity in protein movements and conformational changes. An example of the impact of an R to C change can be found in the insulin receptor. The IR^R252C^ mutation results in an impairment of insulin binding, with downstream effects of reduced insulin degradation and insulin-induced receptor down-regulation [51].

In order to better interpret the functional consequences of the R650C mutation in the LPK rats, we determined that the position of the mutation occurred within a repeated domain found in proteins with so-called propeller structures [36]. This RCC1 domain and the serine/threonine kinase domain are both evolutionary conserved features of the Nek8 protein [36]. The RCC1 and serine/threonine kinase domains could be clearly identified in the predicted 3D structure that was modeled from the amino acid sequence.

Manual comparison to the crystal structure of the Golden hamster RCC1 protein [39] confirmed that the motif in which the R650C mutation occurred is prominent in protein-protein interactions. For example, the outer regions of the blades of the reference RCC1 proteins form the conformationally diverse loop region that interacts with the GTPase Ras-related nuclear protein (Ran) [52]. Another example of an RCC1 domain-containing protein that has a mutation within the G[RCQ]LG sequence is the protein Alsin, a guanine nucleotide exchange factor for Rab5 and Rac1. Mutations in this gene result in autosomal recessive motor neuron disease [53]. Alsin, similar to Nek8, has been predicted to form a 7 bladed propeller. The mutation G540E in Alsin is similar to the H425Y and R650C in Nek8 in that it lies between strands C and D and positions itself within the conserved motif between the R650C and H425Y mutation (G[R650C]L[G540E][H425Y]). Structural analysis by Soares et. al. (2009) [53] predicts that this mutation destabilizes the structure resulting in the observed protein delocalization and cytotoxicity. This is also in accordance with Mollinari et. al (2003) [54] who illustrate the motif as a structural component that hinges the blades together.

A recent study has shown that correct localization of Nek8 to centrosomes and cilia requires both Nek8 catalytic activity and the RCC1 domain [55]. The authors propose that a region incorporating the G448V and H425Y mutations, present within blade 3 of the RCC1 domain, is a centrosome/cilia targeting sequence [55]. The authors demonstrated that none of the known Nek8 mutations identified in NPHP patients, nor that of the *jck* mouse, had altered Nek8 kinase activity. They further propose that autophosphorylation within the RCC1 domain caused a conformational change that reveals a cilia/centrosome-targeting site. Among the Nek8 mutations studied by Zalli et al. (2011) [55] the ones of specific interest were those that were homozygous (ie G448V, H425F). The interpretation of these mutations as exposing a cilia/centrosome targeting motif needs to be modified in light of the R650C mutation reported in this paper. The H425Y mutation and R650C mutations are predicted to be located between strands C and D (blades 3 and 7) and both are located at or near the conserved G[QRC]LG motif. These observations, taken together with other structure function studies on RCC1 like proteins, suggests that the seven bladed beta-propeller structure as a whole is important, rather than individual component blades or part thereof. We propose that the G[QRC]LG motif is an important component for defining the integrity of the ‘axial’ region on which the propeller structure is based.

Taking into account structural similarities of proteins RCC1 domains (Alsin, RCC1 and TD-60 [54]) and the mutations R650C, H425Y and G448V within Nek8’s RCC1 domain, we contend that interactions with different proteins or conformational changes within the propeller structure as a unit *per se* are therefore important in altering the function of Nek8. This model is similar to observations on the RCC1 protein coupled to the GTPase Ran, where all blades of the RCC1 propeller are involved in forming the RCC1-Ran complex [39,52]. Studies on a related protein, Nek9 [56,57] directly defined phosphorylation activity associated with the serine/threonine kinase activity and the control of this activity by the RCC1 domain. Furthermore, it has been demonstrated that the Nek9 RCC1 domain was the domain capable of binding Ran [57,58]. The role of this domain in Nek8 is thus inferred to be a regulatory domain for the kinase.

It is of interest that the mouse and rat mutations in Nek8/NPHP9 lead to a phenotypic presentation of polycystic kidney disease that resembles human ARPKD, with an early onset, enlarged cystic phenotype and widespread cyst development [28,44]. In contrast, in human NPHP, the majority of mutations result in a juvenile onset form of disease, having kidneys that are reduced in size and cysts arising at the corticomedullary border through loss of normal tissue [59]. The notable exception is infantile NPHP, which arises from mutations in NPHP2/Inversin, and presents with a comparable histopathology to that of the rodent models. The known mutations in human Nek8/NPHP9 were identified through screening of patients clinically defined as NPHP, however it is indiscernible from that study what the clinical phenotype of these patients was [10].

Phenotypic difference is not unique in rodent models of PKD, for as noted by Fischer et al (2004) [60], the mouse pcy model of NPHP3 was initially proposed as model of ADPKD [61] and the *pck* rat model, while presenting with an ADPKD phenotype, is due to a mutation in *Pkhd1*, the rat orthologue of the human gene responsible for ARPKD [62]. While the nature of the mutations may be responsible for these phenotypic differences, it has recently been shown that mutations in NPHP2/Inversin always result in infantile NPHP with enlarged kidneys, regardless of the mutation type [63]. Relevant to this study, Inversin and Nek8 have a tightly coupled structural and functional relationship within the cilia [22,64] and future studies are warranted to determine if the interactions between Nek8 and Inversin explain the phenotypic similarities between these forms of NPHP.

## Conclusions

The identification of the LPK mutation in the *Nek8* gene is consistent with many lines of evidence linking this gene to NPHP. The location of the mutation in the RCC1 domain and our demonstration of altered Nek8 localisation supports the hypothesis that ciliary Nek8 plays a key role in normal communication between the cilia and the processes of cellular differentiation and proliferation, such that its dysfunction results in the formation of cysts. Our analysis establishes the LPK rat model for studies aimed at defining the key network systems linking regulation of the cell cycle with cilia, basal bodies and centrosomes, and further, providing a pathological model for the study of cystic kidney disease, and the development of potential intervention and treatment strategies.

## Methods

### Animals

All animals were sourced from the Animal Resource Centre, Murdoch, Western Australia, Australia. Experiments were conducted in accordance with the National Health and Medical Research Council Australian code of practice for the care and use of animals for scientific purposes and approved by the Murdoch University and Macquarie University Animal Ethics Committees.

The *lpk* mutant arose as a spontaneous mutation from the Lewis (LEW/SsNArc) strain at the Animal Resource Centre, (Perth WA, Australia) [28]. Two strategies were used to obtain DNA for linkage genetic analysis. LPK males and Brown Norway (BN/ssArc) females were crossed and first filial (F1) hybrids intercrossed to produce 152 (LPKxBN) second filial (F2) segregants, with a segregation pattern of LPK:non LPK (42:110), as reported previously [28]. LPK and Wistar Kyoto (WKY/NArc) females were crossed and the F1 hybrids crossed back to the LPK males to produce 139 (LPKxWKY) BC1 segregants with a segregation pattern of LPK:non LPK (72:67) within the F2 generation, being consistent with an autosomal recessive mutation.

### Phenotype data

Prior to euthanasia animals were weighed, urine collected and systolic blood pressure determined by tail-cuff plesmography, using an average of 3 measurements after the animals were acclimatized to the procedure (NIBP controller, ADI Instruments, Castle Hill, NSW, Australia). The animals were euthanized using CO_2_/O_2_. Blood was collected by cardiac puncture and tissues were dissected and weighed. Kidneys were then fixed in 4% formalin in 0.01M phosphate buffer (PB) for subsequent histology and the liver was collected and frozen at -80˚C for genomic analysis. Blood was analysed for serum urea and creatinine, total protein and albumin using a Rx Daytona analyser (Randox Laboratories, Antrum, UK). Micro haematocrit tubes were used to determine packed cell volume and urinary protein to creatinine ratio was determined using a Cobas Mira analyser (Roche Diagnostics, Schweiz, AG).

In order to determine the ratio of cyst area to total kidney area, kidney sections were processed for histology by paraffin embedding, sectioning (4μm) and staining with haematoxylin and eosin (H&E). Images of kidney were captured using a Nikon Dx40 Digital SLR and then digitally enhanced to maximize contrast (Additional file 4: Figure S3). The area of cyst to cross sectional area of the kidney (μm^2^) was determined using Image J [65].

### Genotyping

DNA was extracted from the liver using the Wizard® Genomic DNA Purification Kit (Promega, Maddison, WI, USA) following the manufacturer’s protocol. The samples were assayed for DNA concentration and contamination using a NanoDrop 1000 Spectrophotometer (ThermoFisher Scientific, Scoresby, Vic, Australia). DNA was stored at -80˚C and working samples diluted to 50ng/μl in MilliQ H_2_O and stored at -20˚C. Preliminary polymerase chain reaction (PCR) studies of the BC1 using 2-4 simple sequence length polymorphism (SSLP) markers per chromosome (obtained from [66]) mapped the *lpk* locus to chromosome 10 between markers *D10Rat43* (23.42Mbp) and *D10Rat26* (77.09Mbp). Using primer sequences available from Rat Genome Database (RGD) [32] additional polymorphic DNA markers (17 in the BC1 and 12 in the F2, refer Additional file 5: Table S2), synthesized by Geneworks (Adelaide, SA) were then used to further genotype the animals by PCR. The defining markers obtained from the interval mapping from the BC1 and F2 generation were used for comparative analysis between the rat, mouse and human using the Virtual Comparative Map [VCM, RGD [32]].

PCR was performed with Taq polymerase 0.055 U/μl (Fisher Biotech, Australia) in 10 μl reaction volume containing forward and reverse primers 0.5μM, dNTPs 0.25mM, MgCl 1.5mM. Cycling conditions consisted of 4 stages. Stage 1: TAQ activation 94˚C, 5 mins. Stage 2: touchdown protocol, 12 cycles of 94˚C for 30s, annealing for 45s commencing at 54˚C with a 0.5˚C drop in temperature per cycle, and elongation at 74˚C for 30s. Stage 3: 32-40 cycles of denaturing at 94˚C for 30s, annealing at 48˚C for 45s, and elongation at 74˚C for 30s. Stage 4: 1 cycle as per stage 3 with a 5 minute extension and sample hold at 14˚C. PCRs were performed on an *Applied Biosystems Veriti*™ Thermal Cycler.

Genotyping of the animals was carried out on the Protean II xi | XL cell (BioRad) using T = 8% [monomer] and C = 5% [crosslinker] PAGE gels run at 70-80V for 18-22 hours. Gels were post stained with SYBR® Safe (Invitrogen, Aust) for 30 minutes and photographed with Bio-Vision camera with VISION-Capt image acquisition software (v. 15.06, Vilber Lourmat, Germany). The lanes were scored as Homozygote [WKY (A) in the BC1 and BN (A) in the F2 generation & LPK (B)] or Heterozygote (H) (Figure 1).

### QTL analysis

Coinheritance of the phenotypic parameters with polymorphic DNA markers was analyzed by QTL analysis using Map Manager QTX [31] and WinCart QTL [67]. The map in the present paper focused on chromosome 10 based on earlier work (unpublished). The QTL was determined by cumulative interval mapping (CIM) with forward and reverse regression using model 6 with a window size of 0.5 cM and walk speed of 1 cM. The threshold was determined using permutations, with permutations set to 1000 and significance 0.05.

### SNP identification

DNA was extracted from the liver of three 6-week old male LPK animals and three 6-week old male Lewis genetic controls, derived from the same inbred line from which the mutation arose (LEW/SsNArc) but selected without the mutation, using a DNeasy Blood & Tissue Kit (QIAGEN, Vic, Australia) following the manufacturer’s protocol. DNA was checked for quality on a 2% agarose gel and LPK and Lewis samples were respectively pooled.

A Roche Nimblegen sequence capture array (Roche NimbleGen Inc. Madison, WI, USA) was designed to enhance the region of interest and Illumina sequencing reactions of the ~200 bp DNA regions were carried out by the Australian Genome Research Facility Ltd (AGRF, Gehrmann Laboratories, St Lucia, QLD, Australia) yielding 100 bp single end sequence reads. Alignment of the reads was performed using the open source short read aligner Bowtie (release 0.12.5) [68] and limited to 2 mismatches. Homozygous and heterozygous SNPs were identified using the freeware Package SHORE [69]. Samples were concurrently analyzed by AGRF QLD using CASAVA Software (version 1.7, Illumina Inc. San Diego, CA, USA) to identify SNPs. Both approaches yielded the same result. Biological network analyses were drawn from published literature on PKD and links to cilia and cell division target genes of interest were refined further using the web based programs Search Tool for the Retrieval of Interacting Genes/Proteins [70] Cytoscape [71], Inner Medullary Collecting Duct Proteome Database [72] and Murine Immortalized Cortical Collecting Duct Cell (mpkCCD) Transcriptome Database [73].

### Sequence read archive accession numbers

Ilumina raw sequence data for the Lewis (LEW/SsNArc) wild type strains and LPK mutant were submitted to the NCBI Sequence Read Archive (SRA) under the submission numbers SRA056808 and SRA056807, respectively.

### Direct sequencing of target SNP region

Amplification of the identified *lpk* SNP region within Nek8 was carried out using standard PCR techniques with the forward and reverse primers ( nk-f3: 5^′^ TCA AGA TGG TGA TGG TGG 3^′^) and (nk-r3: 5^′^ TGA TGT CAC CGT GTA AGG 3^′^), respectively. Primers were designed using Primer3 [74] to produce a 173 bp product covering the homozygous SNP identified by direct sequencing. Parental BN, WKY & LPK and selected BC1 progeny (animal numbers 85, 87, 90 and 92) were assayed and product visualized on a 1.6% agarose gel. The product was purified using the Promega Wizard® Gel and PCR cleanup system. Thirty ng of product, as determined by spectrometer (Thermo Fisher Scientific, Scoresby, Vic) was used as the template for sequencing using the forward primer only (nk-f3) , with the BigDye Terminator v3.1 Cycle Sequencing Kit (Applied Biosystems, CA, USA) as per the manufacturer’s instructions. The PCR sequencing reaction consisted of 96˚C for 2 mins, followed by 30 cycles of 96˚C for 10s, 48˚C for 10s and 60˚C for 10s. The product was then purified using an EDTA/NaAc ethanol precipitation technique and samples stored at -20˚C. Sequencing was performed on a capillary sequencer (Applied Biosystems 3730xl DNA Analyzer, CA, USA). The chromatograph calls were aligned and visualised using the software Geneious version 5.5 [75].

### Protein structure prediction of the Nek8 protein

The *Rattus norvegicus* Nek8 protein (NP_ 001099274) was aligned to the homologous proteins of *Homo sapiens* (NP_835464), *Mus musculus* (NP_543125), *Gallus gallus* (XP_415822), *Danio rerio* (NP_620766), *Xenopus tropicalis* (NP_001006906), *Xenopus laeivis* (NP_001090238) *Saccoglossus kowalevskii* (XP_002730582). The alignment was conducted using MAFFT version 6.814b [76] using a Blosum62 scoring matrix with the following parameters: 1.53 for the Gap open penalty and 0.123 for the offset value.

RCC1-like repeats for NP_001099274 (*R. novegicus* Nek8) were analysed by the web based program REP [77]. Secondary protein structure was predicted using the Emboss tool Garnier [78], whilst the tertiary protein structure prediction and 3D modeling were performed using Phyre2 [37] and ESyPred [38].

Preliminary work was conducted based on an initial VYxWG motif that early papers described as fingerprinting RCC1 repeats [39]. This was modified to [VILC]xx[WLFC]G, to account for variation within Nek8 and the RCC1 protein. Sequences for the initial blade alignments using MAFFT were determined using the RCC1 motif and selecting 22 amino acids up stream and 25 amino acids downstream. A final blade was selected, being an intervening sequence between blades (non-canonical blade 2). The 7 predicted blades were then aligned using MAFFT against the known RCC1 blades of the *Mesocricetus auratus* (Golden Hamster [UniProtKB: P23800]) for which a crystal structure is available [39].

### Functional domains

Possible functional sites and domains were determined within the Nek8 protein using the web based program Eukaryotic Linear Motif Resource (ELM: [79]). Possible functional motifs within domains were then refined based on predicted 3-D structure of the Nek8 protein and in particular the outer regions of the folded protein because these were most likely to be involved in protein-protein interactions.

### Conserved G[X]LG motif within the blades of the RCC1 domain

The G[X]LG motif was identified as a key feature of the RCC1 domain of Nek8. In order to increase the specificity of the search, the motif GX(5)GXF(4)G[QRC]LG was used in the web based application Prosite [41]. The proteins containing this sequence were then entered into the web based programme KAAS [80] to examine possible pathway connections/ functionality associated with the sequence.

### Scanning electron microscopy

#### Tissue processing

Four Lewis (standard inbred LEW/CrlBR) and 4 LPK animals aged 10-12 weeks of mixed sex were deeply anaesthetised (sodium pentobarbitone 60 mg/kg i.p.) and transcardially perfused at 110mmHg with heparinized saline (0.2% heparin, Mayne Pharma Pty Ltd, Melbourne, Vic, Australia and 0.9% w/v NaCl2), and then 2.5% (v/v) glutaraldehyde solution (ProSciTech Pty Ltd, Brisbane, QLD, Australia) in 0.1M PB, pH 7.4. Kidneys were dissected, sectioned into coronal slices 1 mm thick, and post-fixed for 24 hours in the same fixative solution. Samples were sectioned into 3 mm x 1 mm wedges, washed in 0.1M PB, post-fixed in 1% (v/v) osmium tetroxide solution, washed in 0.1M PB, dehydrated in graded series of ethanol (30-100%) and critical point dried in a EMITECH K850 Critical Point drier, with CO_2_ as a transition fluid. Kidney fragments were mounted on the aluminum stubs, covered previously with carbon tabs and coated with gold using EMITECH sputter coater K550.

#### Imaging and cilia measurement

Samples were viewed with JEOL JSM- 6480 LA Scanning Electron Microscope (Jeol USA, Inc.) and files stored as jpg. To determine cilia length images were opened using Image J software [65] and cilia length measured using the Freehand tool after calibration of the image scale. Cilia were selected from random fields of distal and collecting tubules. For LPK, total number of cilia measurements was 788 (from each animal average n = 197 ± 6.78) and for Lewis, a total of 159 cilia were measured (from each animal average n = 53.5 ± 30.35). The differences in number of cilia measured from each LPK and Lewis rat was due to cysts, which in the LPK allowed ready access of cilia for SEM from broad fields of cells, while in Lewis samples, cross sections of normal tubules presented only small fields of view and therefore limited numbers of cells with cilia available for assessment. A D’Agostino-Pearson test was used to determine normality of data and a two-tailed Mann–Whitney *T*-test was used for comparison of cilia length between Lewis and LPK. Results are presented as median and quartiles (25^th^/ 75^th^ percentile). All analyses were undertaken using the statistical package Prism 5 for Mac (Version 5.0d, GraphPad Software, La Jolla, USA).

### Nek8 immunohistochemistry

Three 10-week old female LPK and three 10-week old female Lewis rats (LEW/CrlBR) were deeply anaesthetised (sodium pentobarbitone 60 mg/kg i.p.) and transcardially perfused at 110mmHg with heparinised saline followed by 4% formalin containing 0.01M PB (pH 7.4) for 45 minutes. Kidneys were removed and post-fixed for 24 hours in the same fixative at 4°C, then stored in 0.01M PB at 4°C until processed. Kidneys were sectioned coronally, paraffin embedded and 10 μm sections cut and mounted onto Superfrost Plus slides, then deparaffinised with xylene and hydrated in a descending series of graded ethanol solutions. Antigen retrieval was performed with 10 mM citrate buffer pH 6 (Sigma Chemical Co., USA). Buffer was heated to boiling, the samples placed in solution and microwaved for 5 min at 720W power and then allowed to cool to room temperature. Sections were then incubated in blocking solution containing tris phosphate buffered saline solution (TPBS-Tx; 10 mM tris base, 0.01 M PB, 0.9% w/v NaCl, 0.1% v/v Triton X 100 and 0.1% w/v sodium azide, pH 7.4) with 10% v/v donkey serum (Sigma-Aldrich, USA) for 1 hour at 20°C. Primary antibodies were diluted in the blocking solution and samples incubated at 4°C overnight. Experiments were initially performed as either single or double labelled experiments using a polyclonal anti-Nek8 antibody (1:200 [81]), a mouse anti-acetylated α-tubulin antibody (1:5000, T7451, clone 611B-1, Sigma-Aldrich, USA) or mouse anti γ-tubulin (1:500, T5326, clone GTU-88, Sigma-Aldrich, USA). After washing in PBS (3 x 30 min), species-specific fluorescent-labelled secondary antibodies were used to detect primary antibody labeling (donkey anti-rabbit Cy3 [1:500, Jackson Immunoresearch, USA] and donkey anti-mouse Dylight 488 [1:500, Jackson Immunoresearch]). Sections were again washed (3 x 30 min) and coverslips mounted using fluorescent mounting media (Dako, Denmark). Additional experiments were performed in the LPK animals with all three antibodies as a triple label, as γ-tubulin and α-tubulin antibodies identified discrete structural regions (basal body and ciliary shaft, respectively) and could be distinguished by location, despite both being identified by the same secondary anti-mouse Dylight 488 antibody.

The sections were imaged on a Leica TCS SP5 confocal microscope (Leica Microsystems, Wetzlar, Germany), equipped with an argon laser, with excitation and detection wavelengths set for Dylight 488 (excitation 488 detection range 496 to 552 nm) and Cy3 (excitation 514 detection range 555 to 625 nm). For double labelled sections, images were collected using sequential acquisition between stacks. Images were adjusted for brightness and contrast only.

## Abbreviations

BC1, Backcross; BN, Brown Norway; Chr, Chromosome; H&E, Haematoxylin and eosin; LPK, Lewis Polycystic Kidney; LOD, Logarithm of odds; NPHP, Nephronophthisis; NIMA, (Never in mitosis gene a)- related kinase 8 (Nek8); PB, Phosphate buffer; PKD, Polycystic kidney disease; PCR, Polymerase chain reaction; QTL, Quantitative trait locus; Ran, Ras-related nuclear protein; RCC1, Regulator of chromosome condensation 1; F2, Second filial; SNP, Single nucleotide polymorphisms; WKY, Wistar Kyoto.

## Competing interests

The authors declare that they have no competing interests.

## Authors’ contributions

JKM carried out the molecular genetic studies, sequence analysis and drafted the manuscript. RAB contributed to the design and analysis of the molecular genetic studies and provided critical review of the manuscript. AD extracted DNA for sequencing purposes and undertook the Nek8/tubulin immunohistochemical experiments. JOK carried out the electron microscopy. MB contributed to the design of the study and analysis of the molecular genetic studies and review of the manuscript. RA participated in the design of the study, assisted in the undertaking and analysis of the molecular genetic studies and drafted the manuscript. GM planned the linkage analyses studies and reviewed the manuscript. JKP conceived of the study and its design, oversaw the experimental protocols, participated in the DNA extractions and immunohistochemical imaging, partook in the drafting of the manuscript and finalization for submission. All authors read and approved the final manuscript.

## Supplementary Material

Additional file 1 Table S1. Candidate genes. Click here for file

Additional file 2 Figure S1. Ancestral DNA sequence and evolutionary conserved amino acids in the NEK8 protein. Click here for file

Additional file 3 Figure S2. RCC1 domains. Click here for file

Additional file 4 Figure S3. Determination of cyst to kidney ratio. Click here for file

Additional file 5 Table S2. Primer List and genomic location based on rn4 rat assembly. Click here for file
