# Supplementary material for: A novel mutation causing nephronophthisis in the Lewis polycystic kidney rat localises to a conserved RCC1 domain in Nek8
Source: BMC Genomics. 2012 Aug 16;13:393. doi: 10.1186/1471-2164-13-393 (PMC3441220; doi:10.1186/1471-2164-13-393)
Supplement: Additional file 1 — Table S1. Candidate genes. [file 1471-2164-13-393-S1.pdf]

Additional File 1

Table S1: Candidate genes

Genes on chromosome 10 from region 63M to 65.5Mbp, selected as candidate genes for the LPK mutation based on known functional roles.

SYMBOL	RGD_ID	NAME	START	STOP	Basis for candidate selection
RGD1309077	1309077	Similar to putative RNA methyltransferase	63600150	63600936	Hypothetical protein associated with RNA binding methyltransferase activity [1].
Phf12	1305731	PHD finger protein 12	64024504	64074603	Transcriptional repressor of activity. Negative regulation of DNA-dependent transcription [1].
Flot2	70993	Flotillin 2	64081463	64104166	Colocalized with activated GPI-linked cell adhesion molecules at the plasma membrane [1].
Spag5	620152	Sperm associated antigen 5	64313146	64330917	Associated with microtubules, flagella structures and sperm motility [2].
Sdf2	1307274	Stromal cell derived factor 2	64378702	64389195	Associated with endoplasmic reticulum (stress induced unfolding). Highly expressed in fast growing, differentiating and meristematic tissues [3].
Supt6h	1309290	Suppressor of Ty 6 homolog (S. cerevisiae)	64388520	64425858	Stimulates transcription elongation [4].
Proca1	1561727	Protein interacting with cyclin A1	64426828	64440614	Protein interacting with cyclin A1-CDK2 complex [5].
Nek8	1306897	NIMA (never in mitosis gene a)- related kinase 8	64458887	64470059	NPHP9 linked mutation (*jck* mouse). Localised to the proximal segment of the cilia inversion compartment. [6, 7].

SYMBOL	RGD_ID	NAME	START	STOP	Basis for candidate selection
Traf4	1306708	Tnf receptor associated factor4	64469647	64477810	TNF associated receptor protein associated with signal transduction developmental neural crest. Linked to epithelial homeostasis and cell polarity [8, 9].
Sarm1	1310078	Sterile alpha and TIR motif containing 1	64584101	64607488	Down regulates NF-kappaB and IRF3 mediated TLR3 and TLR4 signalling. May also directly Inhibit MAPK phosphorylation [10].
Ift20	1309400	Intraflagellar transport 20 homolog (Chlamydomonas)	64647046	64652539	Cilia associated protein linked to PKD cystic kidney (floxed knockout mouse). Increases canonical Wnt signaling. Has basal body & Golgi complex interaction with polycystin 2, kinesin2, Kif3a [11-13].
Nlk	1561602	Nemo like kinase	64681465	64804964	Inhibits canonical Wnt pathway regulation, link to usb4 (ubiquitin pathway) [14].
Ksr1	1308105	Kinase suppressor ras 1	65187915	65322237	A molecular scaffold and positive regulator of the Raf/MEK/ERK phosphorylation cascade [15].

References

1. **Rat Genome Database**, http://rgd.mcw.edu.
2. Fitzgerald CJ, Oko RJ, van der Hoorn FA: **Rat Spag5 associates in somatic cells with endoplasmic reticulum and microtubules but in spermatozoa with outer dense fibers**. *Mol Reprod Dev* 2006, **73**(1):92-100.
3. Schott A, Ravaud S, Keller S, Radzimanowski J, Viotti C, Hillmer S, Sinning I, Strahl S: **Arabidopsis stromal-derived Factor2 (SDF2) is a crucial target of the unfolded protein response in the endoplasmic reticulum**. *J Biol Chem* 2010, **285**(23):18113-18121.
4. Endoh M, Zhu W, Hasegawa J, Watanabe H, Kim DK, Aida M, Inukai N, Narita T, Yamada T, Furuya A *et al*: **Human Spt6 stimulates transcription elongation by RNA polymerase II in vitro**. *Mol Cell Biol* 2004, **24**(8):3324-3336.
5. Diederichs S, Baumer N, Ji P, Metzelder SK, Idos GE, Cauvet T, Wang W, Moller M, Pierschalski S, Gromoll J *et al*: **Identification of interaction partners and substrates of the cyclin A1-CDK2 complex**. *J Biol Chem* 2004, **279**(32):33727-33741.

6. Otto EA, Trapp ML, Schultheiss UT, Helou J, Quarmby LM, Hildebrandt F: **NEK8 mutations affect ciliary and centrosomal localization and may cause nephronophthisis**. *J Am Soc Nephrol* 2008, **19**(3):587-592.

7. Shiba D, Manning DK, Koga H, Beier DR, Yokoyama T: **Inv acts as a molecular anchor for Nphp3 and Nek8 in the proximal segment of primary cilia**. *Cytoskeleton (Hoboken)* 2010, **67**(2):112-119.

8. Kalkan T, Iwasaki Y, Park CY, Thomsen GH: **Tumor necrosis factor-receptor-associated factor-4 is a positive regulator of transforming growth factor-beta signaling that affects neural crest formation**. *Mol Biol Cell* 2009, **20**(14):3436-3450.

9. Kedinger V, Alpy F, Baguet A, Polette M, Stoll I, Chenard MP, Tomasetto C, Rio MC: **Tumor necrosis factor receptor-associated factor 4 is a dynamic tight junction-related shuttle protein involved in epithelium homeostasis**. *PLoS One* 2008, **3**(10):e3518.

10. Peng J, Yuan Q, Lin B, Panneerselvam P, Wang X, Luan XL, Lim SK, Leung BP, Ho B, Ding JL: **SARM inhibits both TRIF- and MyD88-mediated AP-1 activation**. *Eur J Immunol* 2010, **40**(6):1738-1747.

11. Follit JA, San Agustin JT, Xu F, Jonassen JA, Samtani R, Lo CW, Pazour GJ: **The Golgin GMAP210/TRIP11 anchors IFT20 to the Golgi complex**. *PLoS Genet* 2008, **4**(12):e1000315.

12. Jurczyk A, Gromley A, Redick S, San Agustin J, Witman G, Pazour GJ, Peters DJ, Doxsey S: **Pericentrin forms a complex with intraflagellar transport proteins and polycystin-2 and is required for primary cilia assembly**. *J Cell Biol* 2004, **166**(5):637-643.

13. Baker SA, Freeman K, Luby-Phelps K, Pazour GJ, Besharse JC: **IFT20 links kinesin II with a mammalian intraflagellar transport complex that is conserved in motile flagella and sensory cilia**. *J Biol Chem* 2003, **278**(36):34211-34218.

14. Thorpe CJ, Moon RT: **nemo-like kinase is an essential co-activator of Wnt signaling during early zebrafish development**. *Development* 2004, **131**(12):2899-2909.

15. Razidlo GL, Kortum RL, Haferbier JL, Lewis RE: **Phosphorylation regulates KSR1 stability, ERK activation, and cell proliferation**. *J Biol Chem* 2004, **279**(46):47808-47814.
