# Supplementary material for: A novel mutation causing nephronophthisis in the Lewis polycystic kidney rat localises to a conserved RCC1 domain in Nek8
Source: BMC Genomics. 2012 Aug 16;13:393. doi: 10.1186/1471-2164-13-393 (PMC3441220; doi:10.1186/1471-2164-13-393)
Supplement: Additional file 2 — Figure S1. Ancestral DNA sequence and evolutionary conserved amino acids in the NEK8 protein. [file 1471-2164-13-393-S2.PDF]

Additional file 2: Figure S1.

MEKYERIRVVGRGAFGIVHLCLRKADQKLVIIKQIPVEQMTKDERLAAQNECQVLKLLSHPNVIEYYENFLEDKALMIAMEYAPGGTLAEYIQK
RCNSLLDEDTILHFFVQILLALHHVHTKLILHRDLKTQNILLDKHRMIVKIGDFGISKILSSKSKAYTVVGTPCYISPELCEGKPYNQKSDIWALGCV
LYELASLKRAFEAANLPALVLKIMSGTFAPISDRYSPELRQLILSMLNLDPSKRPQLNEIMAQPICIRPLLNLYTDVGSVKMRRVEKPLAPVPTVP
HGRTGSRISSARSRGVRRGPARPGIPPPLSSVYTWGSGISTPLRLPMLNTEVVQVSAGRTQKAGVTKSGRLIMWEAPPMGTAGGPSLPGAV
EQLQPQFISRFLEGQSGVTIKHVSCGDLFTACLTDRGIIMTFGSGSNGCLGHGNFTDVSQPKIVEALLGYEMVQVSCGASHVLAVSNEREVFA
WGRGDNGRLGLGTQESHNSPQQVTVPPEHEAQRVVCGIDSSMILTVQNQILACGSNRFNKLGLDRISSAEEPSPEDQVEEAHSFTPVQSAP
LNQEPIVCADIGTAHSAAVTASGQCYTFGSNQHGQLGTNARRNSRVPCLVAGLQGMKVTMVACGDAFTVAIGAEGEVYTWGKGARGRLG
RRDEDTGIPKPVQLEETHPYVVTSVACCHGNTLLAVKP

Ancestral DNA sequence and evolutionary conserved amino acids in the NEK8 protein.

The mutation identified in the present study at R650C is located in one of the repeat motifs within the RCC1 domain as indicated by the red box. Blue text and orange text indicates conserved sequence, grey text indicates non-conserved sequence. The serine/threonine kinase region of the protein (N-terminus) shows evolutionary conservation (blue). The intermediate region of the protein shows non-conserved regions (grey), while the C-terminal shows a mixed pattern (grey and blue). The orange text highlights the G[QRC]LG motif, which was used in subsequent analysis to identify proteins with a related biological function. The R650C lies within one of these domains. The evolutionary history was inferred using the Maximum Likelihood method based on the JTT matrix-based model in MEGA5 [1].

References

1. Tamura K, Peterson D, Peterson N, Stecher G, Nei M, kumar S: **MEGA5: Molecular Evolutionary Genetics Analysis using Maximum Likelihood, Evolutionary Distance, and Maximum Parsimony Methods**. *Mol Biol Evol* 2011, **28**(10):2731-2739.
