# Supplementary material for: A novel mutation causing nephronophthisis in the Lewis polycystic kidney rat localises to a conserved RCC1 domain in Nek8
Source: BMC Genomics. 2012 Aug 16;13:393. doi: 10.1186/1471-2164-13-393 (PMC3441220; doi:10.1186/1471-2164-13-393)
Supplement: Additional file 3 — Figure S2. RCC1 domains. [file 1471-2164-13-393-S3.pdf]

Additional file 3: Figure S2.

RCC1 Repeat	From	To	Fragment
RCC1-Rep_1	317	355	LSSVYAWGGGLSIPLRLPMMPNTEVVQVAAGRTQKAGVTR
Non_Canonical_2	357	414	GRLILWEAPPLGTGGGTLLPGAVELPQPQFVSRFLEGQSGVTIKHVACGDLFTACLTD
RCC1-Rep_3	415	466	RGIIMTFGSGSNGCLGHGSLTDISQPTIVEALLGYEMVQVACGASHVLALSA
RCC1_Rep_4	467	518	DGELFAWGRGDGGRLGLGTRESHNCPQQVPMVPGQEAQRVVCGIDCSMILTS
RCC1_Rep_5	520	571	GRVLACGSNRFNKLGLDCLSLEEEPVPHPQQVEEALSFTPLGSAPLDRETLLC
RCC1_Rep_6	585	636	SGACYTFGSNQHGQLGTSSRRVSRAPCRVQGLEGIKMVMVACGDAFTVAIGA
RCC1_Rep_7	638	689	GEVYSWGKGARGRLGRRDEDAGLPRPVQLDETHPYTVTSVSCCHGNTLLAVR

RCC1 domains.

Figure illustrates predicted RCC1 domains as defined by the web based program REP (RCC1 repeat, left column) and their alignment around the RCC1 motif (red text), which was used as the basis for three dimensional protein folding predictions. Amino acid positions in the protein are indicated as starting (from) and end (to) positions based on the rat sequence (NP_001099274). Orange bold text highlights the G[QRC]LG motifs, and are also conserved amino acids. An additional RCC1-like repeat domain was annotated as a non-canonical domain as it did not contain the typical repeat motif. Bold green text with box indicates position of the known mutations in Nek8 from human (RCC1-Rep-1,(P), Rcc1-Rep-3 (H) and RCC1-4 (A)) and mouse (RCC1-Rep-3 (G*), jck* and the red square highlight the R650C Nek8 LPK mutation (RCC1-Rep-7 (R).

References

1. Bork P, Andrade MA, Ponting CP, Gibson TJ: **Homology-based method for identification of protein repeats using statistical significance estimates**. *J Mol Biol* 2000, **298**(3):521-537.

2. Otto EA, Trapp ML, Schultheiss UT, Helou J, Quarmby LM, Hildebrandt F: **NEK8 mutations affect ciliary and centrosomal localization and may cause nephronophthisis**. *J Am Soc Nephrol* 2008, **19**(3):587-592.

3. Liu S, Lu W, Obara T, Kuida S, Lehoczky J, Dewar K, Drummond IA, Beier DR: **A defect in a novel Nek-family kinase causes cystic kidney disease in the mouse and in zebrafish**. *Development* 2002, **129**(24):5839-5846.
