# Supplementary material for: A novel mutation causing nephronophthisis in the Lewis polycystic kidney rat localises to a conserved RCC1 domain in Nek8
Source: BMC Genomics. 2012 Aug 16;13:393. doi: 10.1186/1471-2164-13-393 (PMC3441220; doi:10.1186/1471-2164-13-393)
Supplement: Additional file 4 — Figure S3. Determination of cyst to kidney ratio. [file 1471-2164-13-393-S4.pdf]

Additional file 4: Figure S3.

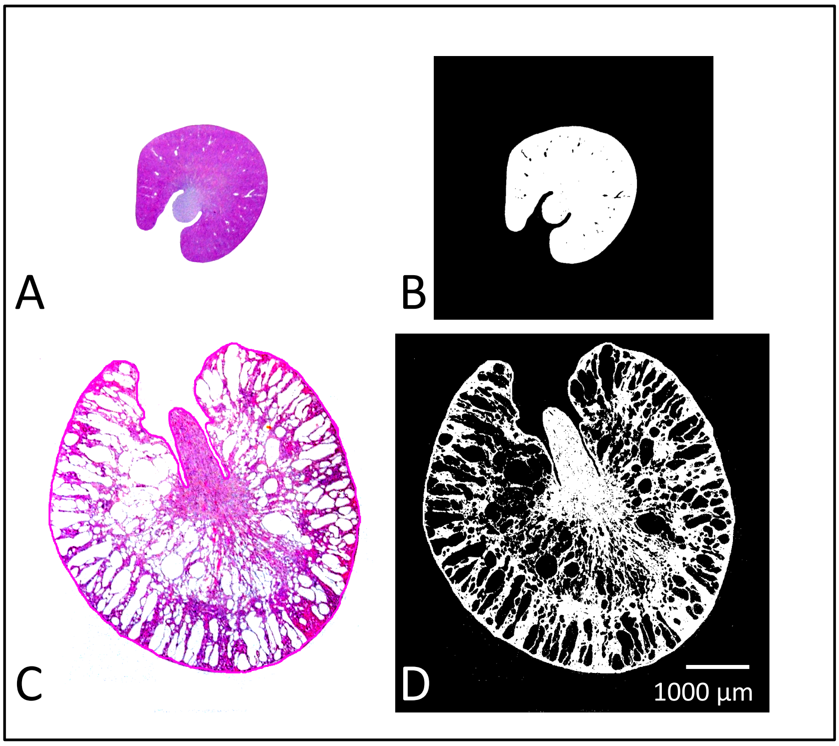

Determination of cyst to kidney ratio.

Kidney sections from a heterozygote BC1 animal (A) and homozygote BC1 animal (C) stained with H&E. Panels B and D show the same respective sections after contrast enhancement to allow determination of cyst area (black) relative to total kidney area (white) within the defined kidney region. The data was then subjected to factor analysis alongside other phenotypic variables using principal components to identify traits with the most predictive power for LPK. Scale bar in panel D is 1000µm for all panels.
