# Supplementary material for: A novel mutation causing nephronophthisis in the Lewis polycystic kidney rat localises to a conserved RCC1 domain in Nek8
Source: BMC Genomics. 2012 Aug 16;13:393. doi: 10.1186/1471-2164-13-393 (PMC3441220; doi:10.1186/1471-2164-13-393)
Supplement: Additional file 5 — Table S2. Primer List and genomic location based on rn4 rat assembly. [file 1471-2164-13-393-S5.PDF]

Additional File 5

Table S2: Primer List and genomic location based on rn4 rat assembly

Primer BC	START bp	Primer F2	START bp
D10Rat43	23,428,128	D10Rat43	23,428,128
		D10Rat38	31668581
D10Rat173	37,505,290		
D10Rat104	41,968,132		
		D10Rat83	46860048
		D10Rat77	56897760
D10Rat160	59,053,888	D10Rat160	59053888
D10Rat80	61,798,249	D10Rat80	61,798,249
		D10Rat159	63166992
D10Rat30	63,752,513	D10Rat30	63,752,513
		D10Mit2	64860950
D10Mco16	65,350,890		
D10Mgh14	65,379,851		
D10Got82	65,996,480		
		D10Rat161	67091545
		D10Rat242	67141196
D10Mgh6	67,677,924		
D10Arb27	69,160,380		
D10Rat28	69,969,510		
D10Rat155	70,229,782		
D10Rat58	71,067,496	D10Rat58	71,067,496
D10Rat26	72,105,962	D10Rat26	72,105,962
D10Rat220	72,285,504	D10Rat220	72,285,504
D10Rat57	74,388,383		
D10Rat86	82,474,813		
D10Rat17	95,066,310		
D10Rat12	99,198,839		
D10Rat4	107,033,561		

Table S2

The LPK locus was determined using length polymorphisms between the WKY, BN and Lewis rat strains. The Table shows a list of primers that were used in both the BC and F2 crosses to define the LPK mutation locus. No crossover events were detected between D10Mco16 and D10Rat30 for n=139 in the BC generation. D10Mit2 achieved 100% genotype to phenotype (PKD) linkage. No further primers were found that produced a length polymorphism between D10Rat30 and D10Rat161. The mutation was defined between 63,752,513 and 65,350,890 Mb on Chromosome10. The D10Rat26 primer locations in the Table are based on the Celera assembly while all the others on the rn4 assembly.
